# Diagnostic accuracy of initial serum β-hCG in predicting pregnancy outcomes post-SET in IVF/ICSI cycles: a systematic review and meta-analysis

**DOI:** 10.3389/fendo.2026.1636981

**Published:** 2026-02-26

**Authors:** Quan Wen, Ran Zhang, Yuan Zhu, Yan Ling, Dandan Xiong

**Affiliations:** 1 Department of Obstetrics and Reproductive Health, Jiangxi Provincial People’s Hospital, The First Affiliated Hospital of Nanchang Medical College, Nanchang, China; 2 Blood Transfusion Department, Jiangxi Provincial People’s Hospital, The First Affiliated Hospital of Nanchang Medical College, Nanchang, China

**Keywords:** meta-analysis, pregnancy outcomes, SET, single-embryo transfer, β-hCG

## Abstract

**Background:**

Serum beta-human chorionic gonadotropin (β-hCG) is a prominent indicator of early pregnancy and is crucial for monitoring pregnancies post-*in vitro* fertilization (IVF). Numerous scholarly investigations had delineated the initial serum β-hCG threshold values using receiver operating characteristic (ROC) curves to distinguish between clinical pregnancy and live birth versus pregnancy failure. However, the variability across these investigations raised concerns about the generalizability of their conclusions to the population undergoing single embryo transfer (SET) within IVF/intracytoplasmic sperm injection (ICSI) cycles. Therefore, this study aimed to critically evaluate the diagnostic accuracy of initial serum β-hCG in predicting clinical pregnancy or live birth outcomes post-SET in IVF/ICSI cycles through a rigorous synthesis of published data.

**Methods:**

A comprehensive literature search was conducted in PubMed, Cochrane Library, EMBASE, Web of Science, China National Knowledge Infrastructure (CNKI), and China Biology Medicine disc databases to identify potentially eligible studies published before December 22, 2023. Studies that adhered to the inclusion and exclusion criteria were incorporated into the meta-analysis without any restrictions based on language. The Quality Assessment of Diagnostic Accuracy Studies-2 (QUADAS-2) checklist was utilized to assess the quality of the included studies. Pooled summary estimates, including sensitivity, specificity, and diagnostic odds ratio (DOR), were calculated. Summary receiver operating characteristic curves (SROC) were constructed, and the area under the curve (AUC) was used to evaluate the prognostic performance of initial serum β-hCG on pregnancy outcomes.

**Results:**

The quantitative synthesis (meta-analysis) included 12 studies, comprising 10 unique entities examining the use of initial serum β-hCG for predicting clinical pregnancy post-SET in IVF/ICSI cycles and 11 entities investigating the effectiveness of initial serum β-hCG in predicting live birth following SET in the same cycles. Initial serum β-hCG showed reference informative diagnostic performance in predicting clinical pregnancy with a pooled sensitivity and specificity of 0.91 and 0.89, respectively, a DOR of 65.07, and an AUC of 0.95. For live birth prediction, initial serum β-hCG demonstrated a certain degree of diagnostic capability with a pooled sensitivity and specificity of 0.87 and 0.70, a DOR of 15.07, and an AUC of 0.82.

**Conclusions:**

Our research assessed the diagnostic efficacy of initial serum β-hCG for detecting clinical pregnancy and live birth through a meta-analysis of data from 12 published studies. This study suggested that the initial serum β-hCG levels had a certain predictive value for pregnancy outcomes following SET in IVF/ICSI cycles.

**Systematic Review Registration:**

https://www.crd.york.ac.uk/PROSPERO/, identifier CRD42023493086.

## Introduction

Infertility is a global health problem, affecting 10%–15% of couples of childbearing age (1). *In vitro* fertilization and embryo transfer (IVF-ET) and its derivative techniques have emerged as crucial therapeutic methods for infertility. However, the IVF success rates are confounded by several factors (2). Single embryo transfer (SET) has been shown to be a successful approach in preventing multiple pregnancies while maintaining cumulative live birth rates comparable to those achieved through double embryos transfer (3, 4). To reduce the risks associated with pregnancy complications and neonatal issues, an increasing number of regions and countries advocate for SET strategy (5–7). However, accurately predicting the outcome of SET, particularly in the initial stages post-transfer, is crucial for optimizing treatment strategies and enhancing the success rate.

Human chorionic gonadotropin (hCG), a glycoprotein of alpha and beta subunits, is synthesized by syncytiotrophoblasts in the placenta (8, 9). Serum beta-human chorionic gonadotropin (β-hCG) is a prominent indicator of early pregnancy and is crucial in monitoring pregnancies post-IVF (10, 11). The quantification of serum β-hCG is employed not only for verifying pregnancy but also due to its correlation with the duration and outcome of gestation (12–14). Preimplantation genetic testing (PGT) is an advanced clinical technique utilized to scrutinize genetic variations within embryos fertilized through IVF combined with intracytoplasmic sperm injection (ICSI) via trophectoderm (TE) biopsy (15). Recent studies have shown that TE biopsy would reduce maternal peripheral blood serum β-hCG levels in early pregnancy (8, 16). In many studies assessing the prognostic significance of serum β-hCG levels in the initial trimester regarding pregnancy outcomes, a clear differentiation is often lacking between the various conditions of SET, multiple ET, and PGT (13, 17–19). Regarding the efficacy of serum β-hCG in predicting pregnancy outcomes following SET, the results reported in the current literature are inconsistent, and a systematic, comprehensive evaluation is still lacking.

Numerous scholarly investigations had delineated the initial serum β-hCG threshold values using receiver operating characteristic (ROC) curves, effectively distinguishing between clinical pregnancy and live birth versus pregnancy failure. However, the variability across these investigations raised concerns about the generalizability of their conclusions to the population undergoing SET within IVF/ICSI cycles. Consequently, this study aimed to critically evaluate the diagnostic accuracy of initial serum β-hCG in predicting clinical pregnancy or live birth outcomes post-SET in IVF/ICSI cycles through a rigorous synthesis of the published data.

## Materials and methods

### Search strategy

This study was performed following preferred reporting items for PRISMA guidelines (20), and the protocol was registered in PROSPERO (ID: CRD42023493086). A systematic literature search was conducted in multiple databases, including PubMed, Cochrane Library, EMBASE, Web of Science, China National Knowledge Infrastructure (CNKI), and China Biology Medicine Disc, on December 22, 2023. We applied no language restrictions. The complete search used for PubMed was: ((((β-human chorionic gonadotropin) OR (Beta-human chorionic gonadotropin) OR (β-hCG) OR (Beta-hCG))) AND (((assisted reproductive technology) OR (ART) OR (*in vitro* fertilization) OR (IVF) OR (intracytoplasmic sperm injection) OR (ICSI)))) AND (pregnancy outcomes). We systematically evaluated all potentially eligible studies for inclusion in this meta-analysis, regardless of their primary outcome or language. Additionally, we manually searched the reference lists of each identified primary study to ensure the comprehensive inclusion of all eligible studies.

### Inclusion and exclusion criteria

The inclusion criteria for this study were as follows: (i) prospective or retrospective cohort studies and case-control studies; (ii) the study population comprised individuals who underwent IVF/ICSI cycles, excluding those who received hCG in all luteal phase-support protocols; (iii) measurement of serum β-hCG levels after ET was conducted to predict the occurrence of clinical pregnancy or live birth; (iv) there was sufficient availability of information to construct a 2 × 2 contingency table, which included the true-positive (TP), true-negative (TN), false-positive (FP), and false-negative (FN) test results at specific cutoff values. The exclusion criteria for this study were as follows: research on natural pregnancy, intrauterine insemination, gamete intra fallopian transfer (GIFT), and donor egg cycles. Additionally, studies focusing solely on patients diagnosed with tubal obstructive infertility, polycystic ovary syndrome, autoimmune diseases, or other diseases were excluded. The population in the studies serving as controls for the PGT group was also included in the analysis if they met the aforementioned inclusion and exclusion criteria. Reviews, conference abstracts, case reports, commentaries, experimental animal studies, and studies with insufficient or unavailable data were excluded from the analysis.

### Study selection and data extraction

Two investigators (WQ and ZR) conducted the literature screening and data extraction, and disagreements were resolved via group discussion. The following data were extracted from the included studies: author, publication time, country, study type, inclusion and exclusion criteria, serum β-hCG assay, stage of transferred embryo, days after ET when β-hCG samples were drawn, cycle type, number of cycles, demographic characteristics of the study population (age and BMI), outcomes, cutoff value, TP, FP, FN, and TN. These data could be obtained directly or calculated indirectly based on the data of the original study. Serum β-hCG concentrations are expressed in mIU/mL (converted to mIU/mL using the conversion formula mIU/mL = 1 IU/L = 1 U/L).

### Risk of bias assessment

Review Manager (RevMan) 5.4.1 was used to assess the quality of the chosen studies following the quality assessment of diagnostic accuracy studies (QUADAS-2) checklist (21). Two impartial reviewers evaluated the potential bias in each study, with any discrepancies resolved by a third reviewer. Each study’s bias risk was classified as low, high, or unclear as a function of the patient selection, index test, reference standard, and flow and timing.

### Statistical methods

The statistical calculation was performed using STATA software (version 16.0). The significance level for the statistical analysis of this meta-analysis was set at α < 0.05. After integration, the sensitivity, specificity, positive likelihood ratio (PLR), negative likelihood ratio (NLR), and diagnostic odds ratio (DOR) were calculated. Calculating the area under the curve (AUC) involved plotting summary receiver operating characteristic curves (SROC) to assess the aggregated diagnostic efficacy of serum β-hCG. Heterogeneity within the study was assessed using Cochrane Q and I^2^ statistics. Fixed effects or random-effects models were used according to the heterogeneity (I^2^ statistic > 50%, random effects models; I^2^ statistic < 50%, fixed effects model). In this analysis, the bivariate random-effects model was employed. A Fagan nomogram was employed to calculate the posterior probability. Deek’s funnel plot was used to assess publication bias among the included studies.

## Results

### Study selection and characteristics

**Figure 1:**
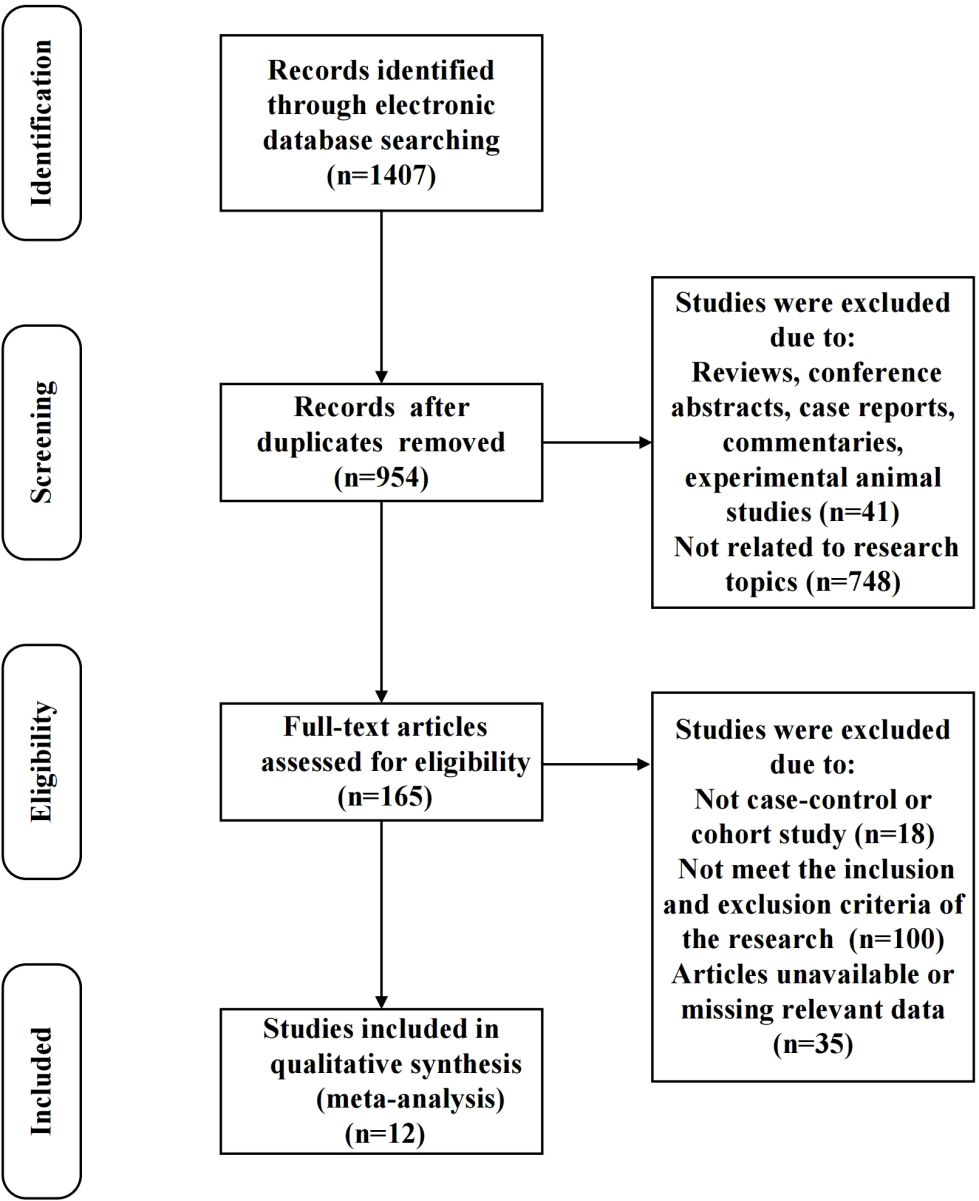
Flowchart of studies included in the meta-analysis.

A detailed flowchart outlining the study selection process was presented in **Figure 1**. We initially identified 1407 records through electronic database searches. After deduplication, 954 unique records were identified for further consideration. During the preliminary screening phase, 789 records were excluded: 41 reviews, conference abstracts, case reports, commentaries, and experimental animal studies, and 748 were unrelated to the research topics. This left 165 studies for full-text review. Then, stringent inclusion and exclusion criteria were applied to ensure the highest relevance and quality of the included studies. Finally, 12 studies were included in the quantitative synthesis (meta-analysis) (8, 16, 22–31).

The attributes of the studies incorporated in the analysis were detailed in **Table 1**, with each study identified as a retrospective cohort study. Except for one study involving patients who underwent a single cleavage embryo or blastocyst transfer (22), all other studies focused on patients who received a single blastocyst transfer. In the included studies, some have delineated distinct cycle types when investigating the impact of β-hCG on clinical pregnancy or live birth outcomes, differentiating between fresh and frozen embryo cycles. For studies that included data from both fresh and frozen embryo cycles and conducted separate analyses for each, we treated them as two separate study entities within our analysis. Accordingly, we labeled them Study 1 and Study 2 to maintain clarity and distinction. Ultimately, 8 studies evaluated serum β-hCG application in predicting clinical pregnancy, with 2 of these studies encompassing different cycle types, totaling 10 study entities. Nine studies explored serum β-hCG efficacy in predicting live birth, with 2 of these considering different cycle types, resulting in 11 study entities. For detailed information, refer to **Tables 2**, **3**.

**Table 1:** Characteristics of the studies included in the meta-analysis.

Study	Year	Country	Study type	Serum β-hCG assay	Value defined as positive for serum β-hCG (mIU/mL)	Days after ET when β-hCG samples drawn	Transferred embryo type	Number of cycles	Age	BMI
Li et al (8)	2022	China	Retrospective cohort	NA	7	12	Frozen blastocyst	5190	32.00 ± 4.44	21.78 ± 2.52
Lu et al (16)	2020	China	Retrospective cohort	NA	10	12	Frozen blastocyst	465	31.83 ± 4.18	20.80 ± 3.70
Al Mamari et al (22)	2019	Canada	Retrospective cohort	Solid-phase two-site chemiluminescent immunometric assay	100	16	Fresh cleavage embryo or blastocyst	1076	NA	NA
Oron et al (23)	2017	Canada	Retrospective cohort	Immunometric sandwich assay	5	11	Fresh blastocyst	789	34.00 ± 3.80	24.60 ± 5.50
							Frozen blastocyst	341	34.20 ± 4.30	24.50 ± 5.30
Ozer et al (24)	2023	Turkey	Retrospective cohort	Chemiluminescence immunoassay	20	9	Fresh blastocyst	738	31.83 ± 4.23	24.41 ± 4.51
							Frozen blastocyst	2500	30.92 ± 4.23	25.04 ± 4.64
Lin et al (25)	2019	China	Retrospective cohort	Chemiluminescence immunoassay	30	14	Frozen blastocyst	1078	30.44 ± 3.84	NA
Qiu et al (26)	2021	China	Retrospective cohort	chemiluminescence-based immunoassay	5	10	Frozen blastocyst	772	30.48 ± 6.50	NA
Wu et al (27)	2021	China	Retrospective cohort	Immunochemiluminometric assay	25	14	Frozen blastocyst	267	30.50 ± 4.10	22.65 ± 8.28
Cai et al (28)	2023	China	Retrospective cohort	Electrochemiluminescence immunoassay	5	12	Frozen blastocyst	519	30.00 ± 4.44	20.8 ± 2.67
Xiong et al (29)	2019	China	Retrospective cohort	Chemiluminescent micro-particle immunoassay technology	5	11	Frozen blastocyst	640	33.79 ± 4.23	21.35 ± 2.93
Zhang et al (30)	2022	China	Retrospective cohort	Electrochemical luminescence	5	14	fresh or frozen blastocyst	4678	31.01 ± 4.57	23.96 ± 3.30
Zhao et al (31)	2017	China	Retrospective cohort	NA	5	12	Fresh blastocyst	214	32.22 ± 4.57	22.07 ± 3.02
							Frozen blastocyst	1513	31.29 ± 4.01	21.68 ± 3.02

NA, not acquired; ET, embryo transfer; β-hCG, beta-human chorionic gonadotropin.

**Table 2:** The contingency table (TP, FP, FN, and TN) of the study entities included in the meta-analysis for predicting clinical pregnancy.

Study ID	Cycle type	Cutoff value (mIU/mL)	TP	FP	FN	TN
Al Mamari	Fresh	190	622	40	144	270
Zhang	fresh or frozen	503	3878	34	319	447
Oron1	fresh	111	574	60	64	91
Oron2	frozen	137	192	35	39	75
Qiu	frozen	113	410	24	23	315
Wu	frozen	302	252	0	7	8
Zhao1	fresh	213	146	5	11	52
Zhao2	frozen	400	1057	73	125	258
Xiong	frozen	152	489	16	36	99
Lin	frozen	630	925	0	90	63

FN, false-negative; FP, false-positive; TN, true-negative; TP, true-positive.

**Table 3:** The contingency table (TP, FP, FN, and TN) of the study entities included in the meta-analysis for predicting live birth.

Study ID	Cycle type	Cutoff value (mIU/mL)	TP	FP	FN	TN
Al Mamari	Fresh	213	213	458	146	114
Lu	frozen	411	411	347	23	42
Li	frozen	299	299	2678	546	328
Qiu	frozen	146	146	329	75	18
Wu	frozen	1621	1621	120	21	64
Ozer1	fresh	117	117	463	52	102
Ozer2	frozen	132	132	1319	205	539
Zhao1	fresh	223	223	99	38	4
Zhao2	frozen	411	411	640	328	68
Xiong	frozen	212	212	351	126	19
Cai	frozen	658	658	298	37	90

FN, false-negative; FP, false-positive; TN, true-negative; TP, true-positive.

### Assessment of study quality

**Figure 2:**
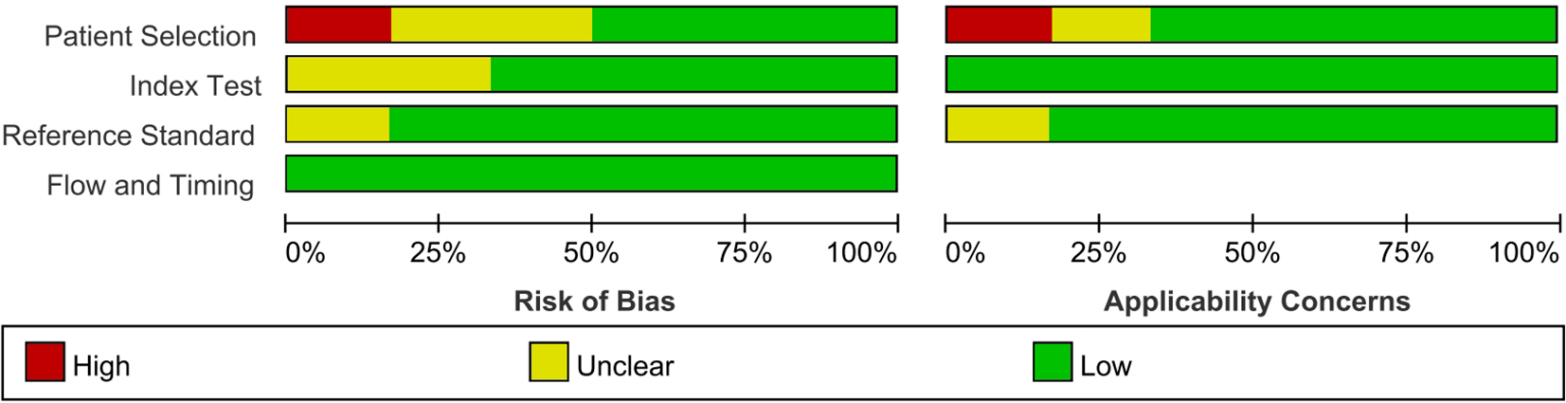
Quality assessment of diagnostic accuracy studies in the meta-analysis. High, high risk; Unclear, unclear risk; Low, low risk.

**Figure 2** showed the QUADAS-2 quality assessment for the 12 included articles. None of the studies fulfilled all the quality criteria. The most common risk of bias was related to patient selection, with two studies (16.7%) exhibiting a high risk of bias, 4 studies (33.3%) presenting an unclear risk of bias, and 6 studies (50.0%) demonstrating a low risk of bias. In the domains of the index test and the reference standard, 4 studies (33.3%) and 2 studies (16.7%), respectively, demonstrated unclear risks of bias. The remaining studies in the two domains showed low risks of bias. Regarding flow and timing, all studies were evaluated as having low risks of bias. In assessing the applicability concerns, two studies (16.7%) exhibited high concern risk, two studies (16.7%) displayed unclear concern risk, and the remaining studies (66.7%) demonstrated low concern risk in patient selection. For the index test, all studies showed a low concern risk. In the reference standard domain, two studies (16.7%) indicated unclear concern risk, while most studies (83.3%) showed low concern risk. Therefore, the included studies demonstrated moderate methodological quality overall.

### Accuracy of serum β-hCG in prediction of clinical pregnancy

**Figure 3:**
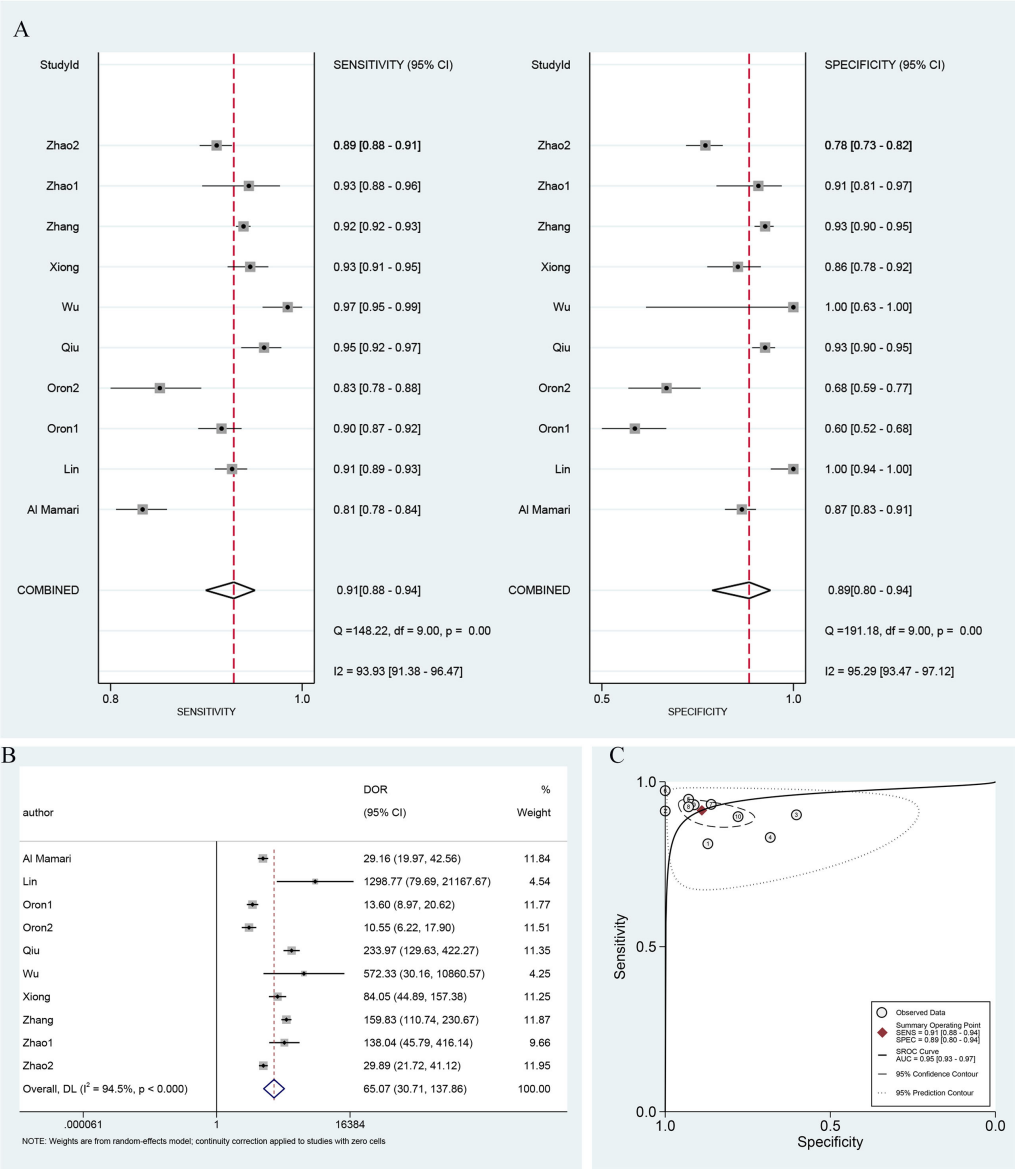
Diagnostic performance metrics for serum β-hCG in clinical pregnancy. **(A)** Forest plot of sensitivity and specificity, **(B)** forest plot of DOR, and **(C)** the SROC curve and pooled AUC value. DOR, diagnostic odds ratio; AUC, area under the curve; and SROC, summary receiver operator characteristic.

A threshold effect test was first conducted, with Spearman correlation analysis indicating no threshold effect (r = -0.539, P = 0.108). Given the considerable heterogeneity observed among studies, a random-effects model was utilized to determine the diagnostic metrics of serum β-hCG in predicting clinical pregnancy. The pooled sensitivity and specificity were calculated to be 0.91 (95% confidence interval [CI]: 0.88–0.94) and 0.89 (95% CI: 0.80–0.94), respectively, with the forest plot depicted in **Figure 3A**. **Figure 3B** illustrated the pooled DOR, which is 65.07 (95% CI: 30.71–137.86). Furthermore, the SROC curve was plotted, revealing a pooled AUC score of 0.95 (95% CI: 0.93–0.97), as depicted in **Figure 3C**. These pooled analysis outcomes collectively highlighted a reference informative diagnostic performance of serum β-HCG in forecasting clinical pregnancy.

To explore the potential sources of heterogeneity, subgroup analyses were conducted. Groups were stratified according to the timing of serum β-hCG measurement (9–10 days, 11–13 days, and 14–16 days after ET), cycle type (fresh vs. frozen), and geographic region (China vs. other countries). The results showed no significant heterogeneity between groups based on measurement timing (p = 0.718) (**Supplementary Figure 1A**) or cycle type (p = 0.209) (**Supplementary Figure 1B**). Although a statistically significant difference was observed between geographic regions (p < 0.001) (**Supplementary Figure 1C**), substantial heterogeneity remained within both subgroups under this classification. Taken together, these findings suggested that none of the three grouping factors served as a major source of heterogeneity in this study.

### Accuracy of serum β-hCG in the prediction of live birth

**Figure 4:**
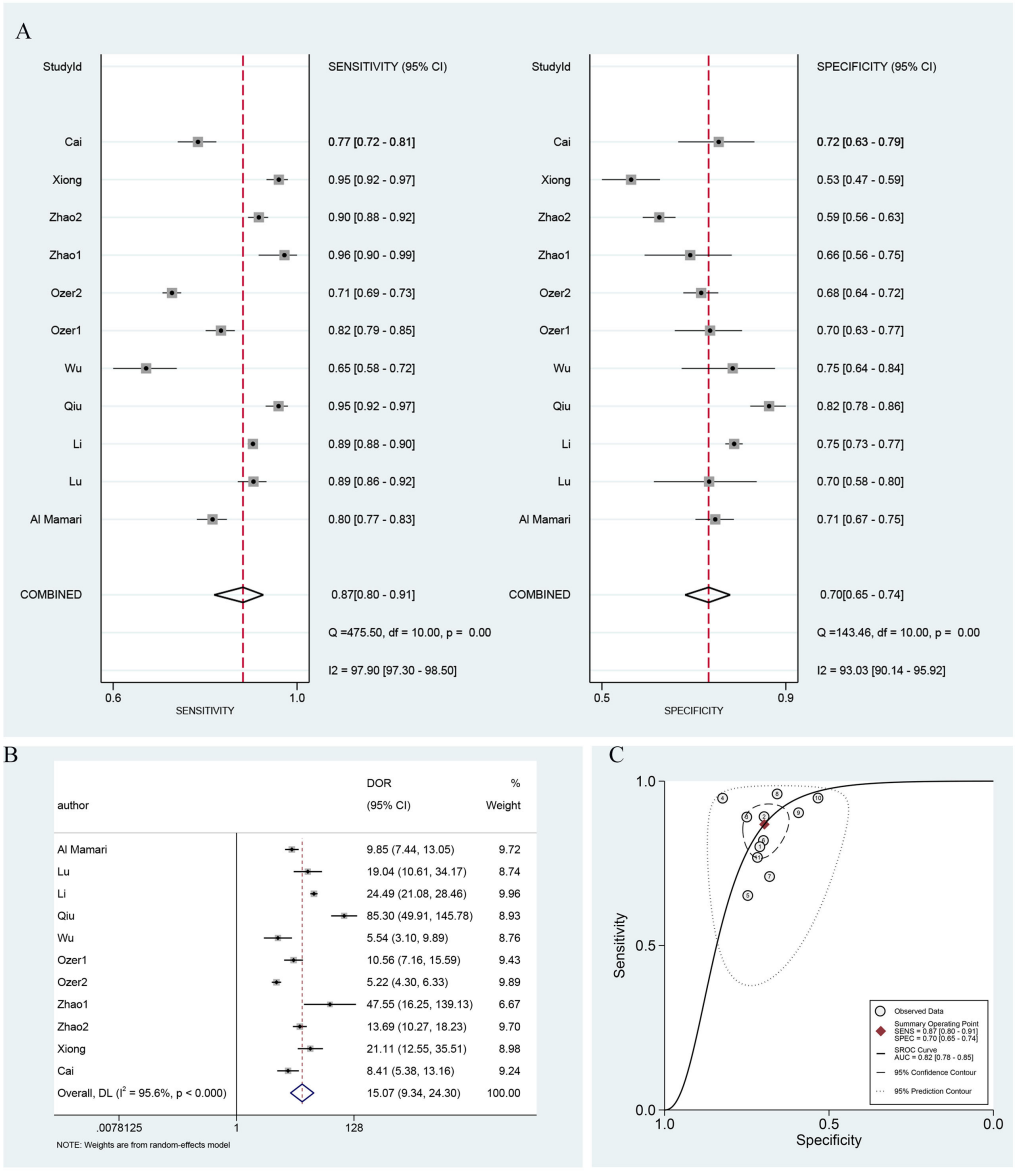
Diagnostic performance metrics for serum β-hCG in live birth. **(A)** Forest plot of sensitivity and specificity, **(B)** forest plot of DOR, and **(C)** the SROC curve and pooled AUC value. DOR, diagnostic odds ratio; AUC, area under the curve; and SROC, summary receiver operator characteristic.

Similarly, Spearman correlation analysis indicated no significant threshold effect for live birth prediction (r = 0.400, P = 0.223). Given the significant variability across studies, the random effects model was again employed to gauge the diagnostic efficacy of serum β-hCG concerning live birth. The pooled sensitivity and specificity were 0.87 (95% CI: 0.80–0.91) and 0.70 (95% CI: 0.65–0.75), respectively, with the corresponding forest plot in **Figure 4A**. **Figure 4B** detailed the pooled DOR, which was 15.07 (95% CI: 9.34–24.30). The SROC curve was also constructed, with the pooled AUC depicted in **Figure 4C** at 0.82 (95% CI: 0.78–0.85). These findings from the pooled analysis indicated that serum β-HCG demonstrates a certain degree of diagnostic capability in predicting live birth.

To explore potential sources of heterogeneity in the prediction of live birth, subgroup analyses were also performed based on the same stratifications. The results showed no significant heterogeneity between groups for measurement timing (p = 0.056) (**Supplementary Figure 1D**) or cycle type (p = 0.747) (**Supplementary Figure 1E**). Although a statistically significant difference was observed between geographic regions (p = 0.012) (**Supplementary Figure 1F**), notable heterogeneity persisted within both subgroups. Therefore, these factors were also not identified as major sources of heterogeneity for live birth prediction.

### Publication bias

The potential for publication bias in research on the utility of β-hCG in forecasting clinical pregnancy and live birth was rigorously evaluated using Deek’s funnel plot asymmetry test. **Supplementary Figure 2** presented the results, which were notably free from bias. The corresponding P-values for the tests were 0.91 and 0.74, respectively, both comfortably surpassing the threshold of 0.05, highlighting robust integrity in the publication process, with no discernible publication bias detected.

### Clinical significance of the study

**Figure 5:**
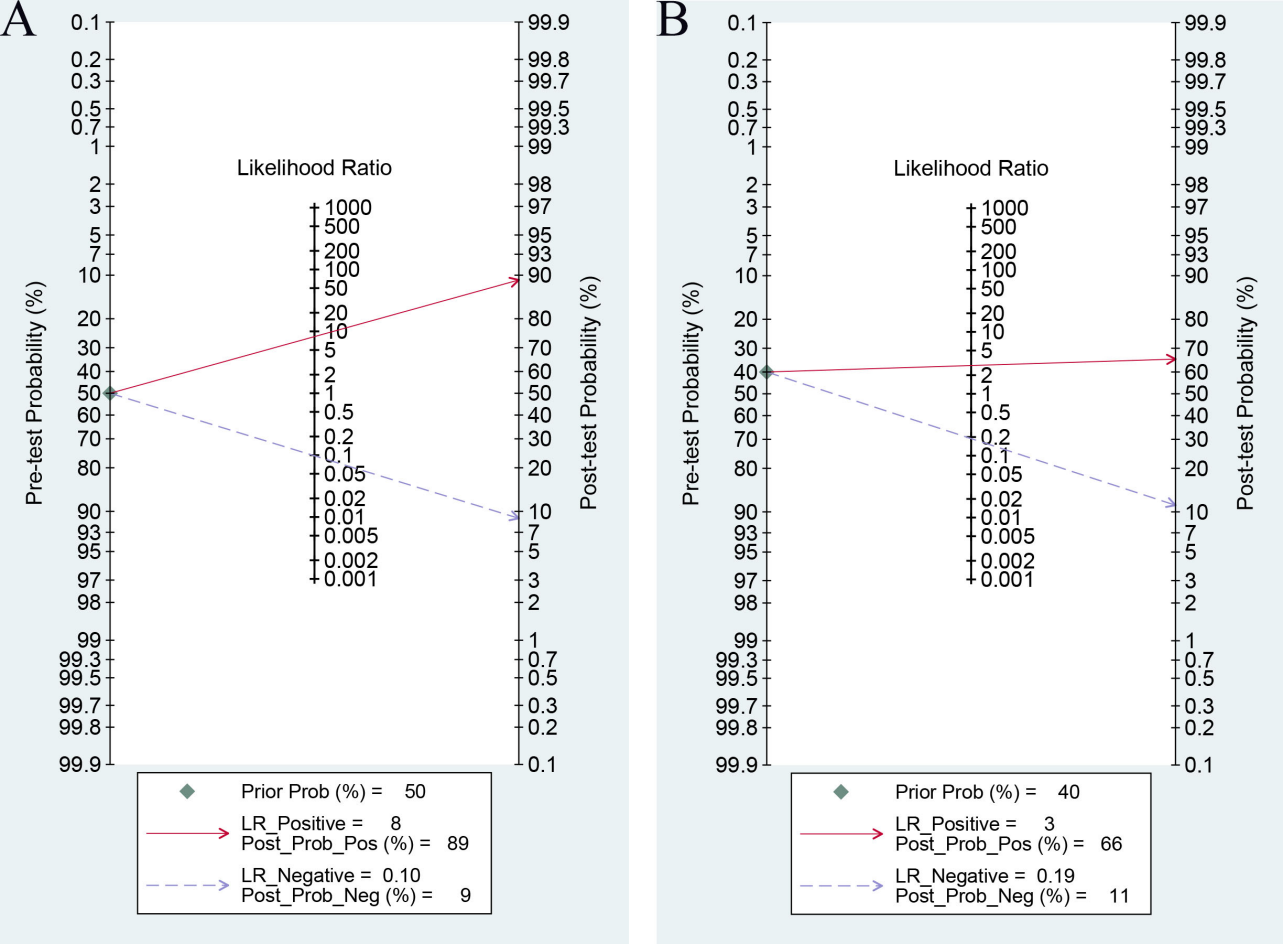
Fagan plot of serum β-hCG for predicting clinical pregnancy **(A)** and live birth **(B)**.

In clinics, physicians expect to utilize initial serum β-hCG levels to forecast pregnancy outcomes after ET, delivering personalized medical advice to enhance the probability of a healthy pregnancy and live birth. This approach also aids in clarifying outcomes for patients and alleviating their uncertainty and anxiety. To elucidate the clinical relevance of our findings, we constructed a Fagan plot to delineate the relationship between pretest probability, likelihood ratio, and post-test probability. **Figure 5A** illustrated that with a pretest probability of 50%, a positive diagnostic likelihood ratio of 8 elevates the post-test probability to 89%, underscoring the substantial clinical diagnostic utility of serum β-hCG for confirming clinical pregnancy. Similarly, **Figure 5B** demonstrated that for a pretest probability of 40%, a PLR of 3 results in a post-test probability of 66%, highlighting the serum β-hCG’s value in predicting live birth.

**Figure 6:**
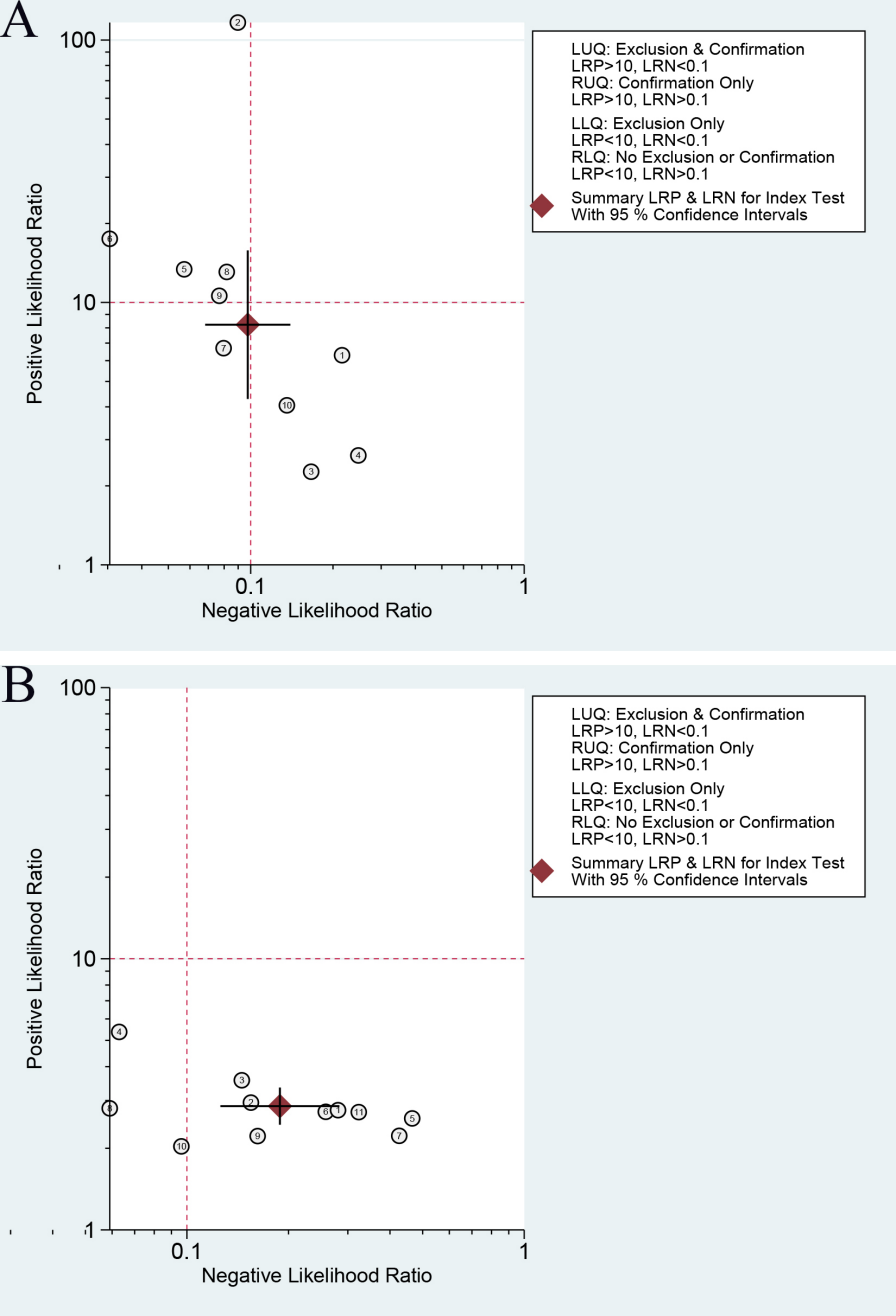
The likelihood ratio scattergram to estimate the clinical significance in clinical pregnancy **(A)** and live birth **(B)** diagnosis of serum β-hCG.

In our analysis, the likelihood ratio scattergram provided insightful distribution patterns for the predictive power of serum β-hCG across different clinical outcomes. When predicting clinical pregnancy, the included studies were predominantly found in the first (left upper, LUQ), second (left lower, LLQ), and fourth (right lower, RLQ) quadrants (**Figure 6A**). When predicting live birth, most studies concentrated on the RLQ (**Figure 6B**). These findings indicated that the diagnostic utility of serum β-hCG is restricted in confirming or excluding clinical pregnancy and live birth.

## Discussion

For clinicians to assess early pregnancy viability, an increase in serum β‐hCG level is crucial if ultrasonography is non-diagnostic (32). To our knowledge, this is the first systematic review and meta-analysis of initial serum β-hCG in predicting clinical pregnancy and live birth outcomes post-SET in IVF/ICSI cycles. All studies included in this systematic review were published within seven years, indicating significant recent attention and heightened interest and concern within the academic community.

This systematic review synthesized data from 12 studies, comprising 10 study entities focused on predicting clinical pregnancy and involving 11,368 cases. Furthermore, the review encompassed 11 study entities dedicated to predicting live birth outcomes, analyzing data from 13,894 cases. The studies included were assessed for quality and determined to be of a relatively high standard, with no evidence of publication bias, thereby enhancing the findings’ reliability. The results of the pooled analysis suggested a favorable diagnostic efficacy of serum β-hCG for both clinical pregnancy and live birth outcomes post-SET in IVF/ICSI cycles.

Several systematic reviews and meta-analyses had been published regarding the association between hCG levels and adverse pregnancy outcomes in recent years. Huang et al. (33) discovered that elevated levels of β-hCG in singleton women during pregnancy are correlated with an increased likelihood of experiencing pregnancy complications and adverse outcomes. Skogler et al. (34) conducted a meta-analysis that suggested a potential correlation between elevated hCG levels, measured as multiples of the median at or above 2.0/2.3/2.5, and an increased likelihood of preeclampsia (OR 2.08, 95% CI: 1.26–3.44) and preterm delivery (OR 1.29, 95% CI: 1.12–1.47). Nevertheless, the certainty of this association remains uncertain. The analysis encompassed studies that assessed maternal blood hCG levels during the first and second trimesters, with normalization of hCG levels to the median for the corresponding gestational age. Peris et al. (35) found that abnormal first-trimester hCG levels are associated with higher risks of fetal demise *in utero* and preeclampsia, possibly indicating placental dysfunction. The study also noted a minor link between hCG levels and outcomes, such as gestational diabetes, preterm birth, and placental abruption. Galperin et al. (10) found that the initial hCG level on day 28 after IVF was a more accurate predictor of live birth than that on day 31. At this stage, an hCG level of 49.2 IU/L correctly identified 93.7% of those who would have a live birth, while a level of 108 IU/L identified 71.5%. Nevertheless, our study focused on the diagnostic accuracy of initial serum β-hCG in predicting clinical pregnancy or live birth outcomes.

The effectiveness of initial serum β-hCG levels in predicting pregnancy outcomes varies across different research studies. Eskandar et al. demonstrated that the initial serum β-hCG showed limited predictive accuracy in identifying patients with ongoing pregnancy and live birth, as evidenced by sensitivities of 78.7% and 77.9%, specificities of 75.0% and 61.6%, and AUC values of 0.63 and 0.58, respectively (18). Dahiya et al.’s research found that at 17 days post-oocyte retrieval, a serum β-hCG level of 199 IU/L predicted live birth with 69.5% sensitivity and 50% specificity in cleavage-stage transfers (AUC 0.62), while a level of 253 IU/L exhibited 61.2% sensitivity and 63.6% specificity for blastocyst transfers (AUC 0.75) (17). The accuracy of predictive outcomes may be influenced by various factors, including the number of embryos transferred, the type of cycle, the developmental stage of the transferred embryos, and the timing of β-hCG sample collection following embryo transfer. When only SET was considered, our results revealed that initial serum β-hCG displayed a high diagnostic accuracy for predicting both clinical pregnancy and live birth outcomes in IVF/ICSI cycles.

However, when interpreting the favorable diagnostic performance of initial serum β-hCG as reported in this meta-analysis, it is essential to situate it within a broader prognostic context. While the findings of this study confirm its significant value as a post-transfer biomarker, contemporary understanding of pregnancy outcome prediction in ART, particularly under the SET strategy, places greater emphasis on the multifactorial nature of such predictions. Successful pregnancy outcomes are influenced by a combination of peri-cycle parameters and baseline reproductive indicators, rather than relying solely on a single post-transfer biomarker. Studies have shown that the progesterone to number of mature oocytes index (PMOI) on the day of hCG injection can independently and effectively predict pregnancy outcomes in fresh IVF/ICSI cycles (36). Additionally, baseline markers of ovarian reserve and responsiveness, such as anti-Müllerian hormone (AMH) and antral follicle count (AFC), as well as factors like age and oocyte yield, have been demonstrated to hold predictive value for clinical pregnancy outcomes (37, 38). Thus, outcome prediction in IVF/ICSI exhibited a distinctly multidimensional characteristic, with initial serum β-hCG representing only one important component within this comprehensive predictive framework.

Furthermore, the forest plot analysis revealed significant heterogeneity across the studies, and subgroup analysis did not identify the sources of this heterogeneity. This may be attributable to complex or unquantified factors, including variations in population characteristics—such as differences in age, infertility etiology, and embryo quality—as well as discrepancies in study design and methodology, such as whether studies were prospective, the use of blinding, and the level of procedural standardization. This heterogeneity constitutes an important limitation in interpreting the present findings. The included studies employed various serum β-hCG detection methods (e.g., chemiluminescence, immunometric, electrochemical assays). Differences among these methodologies may lead to variations in absolute measured values, thereby affecting comparability across studies. Additionally, substantial differences existed among studies in terms of cutoff values and the timing of serum β-hCG measurement. Without standardizing or stratifying these cutoffs, directly pooling diagnostic accuracy data could lead to overestimation of the summary AUC. Therefore, based on the current evidence, it was not yet possible to recommend a universal cutoff value. Therefore, future studies with rigorous designs and standardized definitions should be conducted to establish a stronger evidence base.

Additionally, in this analysis, the pretest probability for clinical pregnancy was set at 50%, and for live birth at 40%. This value did not reflect the actual prevalence in any specific population but was chosen to avoid bias that might arise from selecting a single fixed value, given the considerable variation in baseline characteristics and outcome rates across studies. In clinical practice, it is recommended that clinicians recalculate predictive values tailored to individual patient contexts—incorporating factors such as age, embryo quality, and actual pregnancy or live birth rates—to facilitate the translation of these findings into personalized clinical decision-making.

In assisted reproductive technology, as the SET strategy is increasingly advocated, clinical practice needs to establish corresponding and more precise standards. Our results revealed that establishing a serum β-hCG prediction value for patients undergoing SET can enhance the accuracy of predictive outcomes, providing clinical physicians with reference information for early intervention in patients and aiding in alleviating patients’ anxiety promptly after embryo transfer. Looking forward, to fully realize the potential of precision medicine in reproductive endocrinology, future research should focus on developing and validating combined or multivariable predictive models. These models would integrate baseline characteristics, key cycle-specific parameters, and post-transfer biomarkers. Particularly for the SET population, these comprehensive models are expected to enable more accurate individualized prognostic assessments, thereby optimizing clinical decision-making and personalized patient management strategies.

## Conclusions

Our research assessed the diagnostic efficacy of initial serum β-hCG for detecting clinical pregnancy and live birth through a meta-analysis of data extracted from 12 published studies. This study suggested that the initial serum β-hCG levels had a certain predictive value for pregnancy outcomes following SET in IVF/ICSI cycles. However, due to potential spectrum bias, this conclusion warranted cautious interpretation. While serum β-hCG served as a useful supportive prognostic marker, it was currently insufficient to function as a standalone clinical decision-making tool.

## Date availability statement

The original contributions presented in the study are included in the article/**Supplementary Material**. Further inquiries can be directed to the corresponding author.

## Supplementary material

Supplementary Figure 1 Forest plot showing pooled odds ratios of subgroup analyses for serum β-hCG in predicting clinical pregnancy (panels **A–C**) and live birth (panels D–F). Subgroups were defined as follows: timing of serum β−hCG measurement (panels A and D: 0 = 9–10 days, 1 = 11–13 days, 2 = 14–16 days after ET), cycle type (panels B and E: 0 = fresh, 1 = frozen), and geographic region (panels C and F: 1 = China, 0 = other countries).

Supplementary Figure 2 Deeks funnel plot. **(A)** Assessing publication bias in study entities evaluating clinical pregnancy and **(B)** assessing publication bias in study entities evaluating live birth.
