# Supplementary figures and images for: Diagnostic accuracy of initial serum β-hCG in predicting pregnancy outcomes post-SET in IVF/ICSI cycles: a systematic review and meta-analysis
Source: Front Endocrinol (Lausanne). 2026 Feb 26;17:1636981. doi: 10.3389/fendo.2026.1636981 (PMC12979143; doi:10.3389/fendo.2026.1636981)

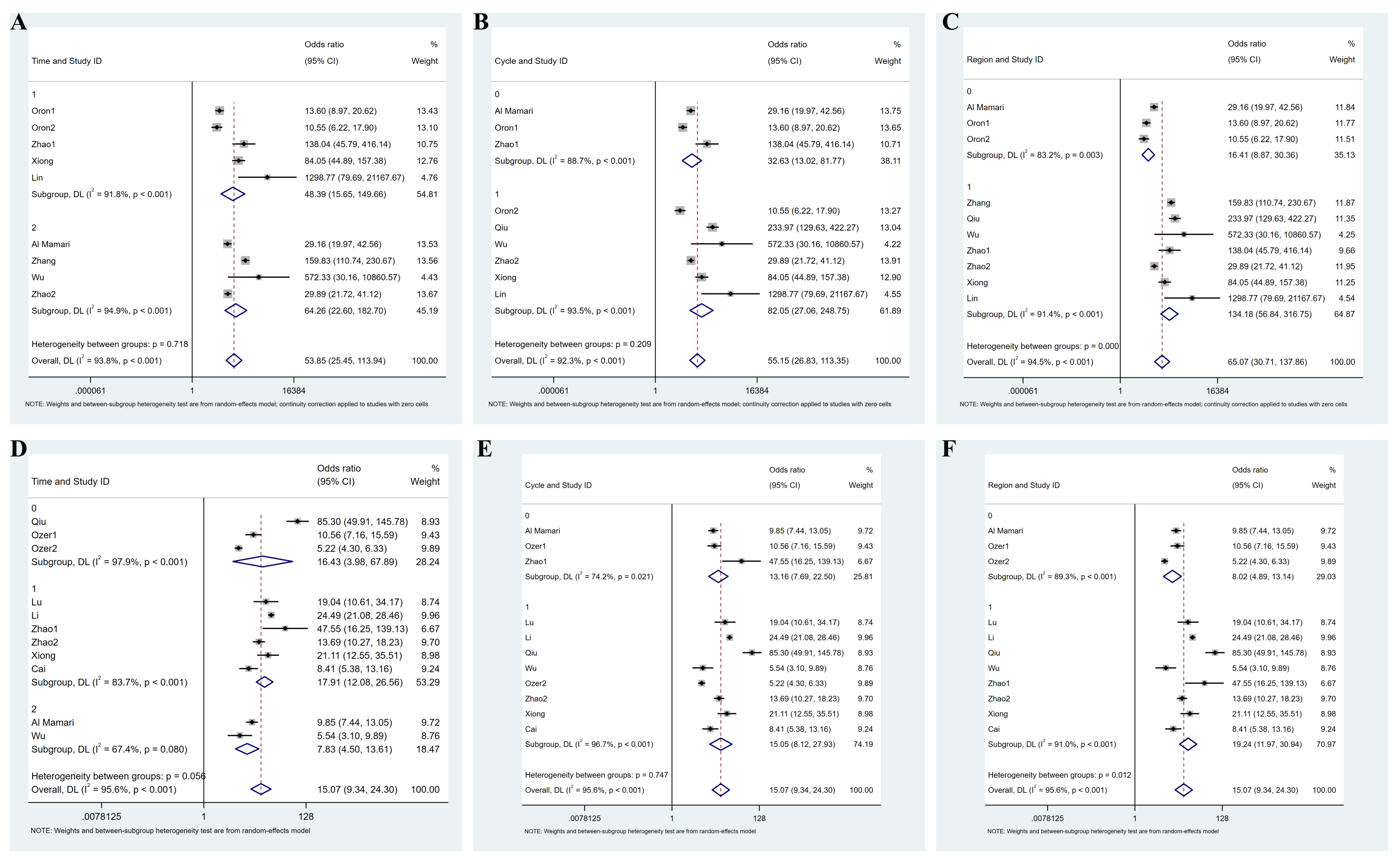

Supplement: Supplementary Figure 1 — Forest plot showing pooled odds ratios of subgroup analyses for serum β-hCG in predicting clinical pregnancy (panels A–C) and live birth (panels D–F). Subgroups were defined as follows: timing of serum β−hCG measurement (panels A and D: 0 = 9–10 days, 1 = 11–13 days, 2 = 14–16 days after ET), cycle type (panels B and E: 0 = fresh, 1 = frozen), and geographic region (panels C and F: 1 = China, 0 = other countries). [file Image1.tiff]

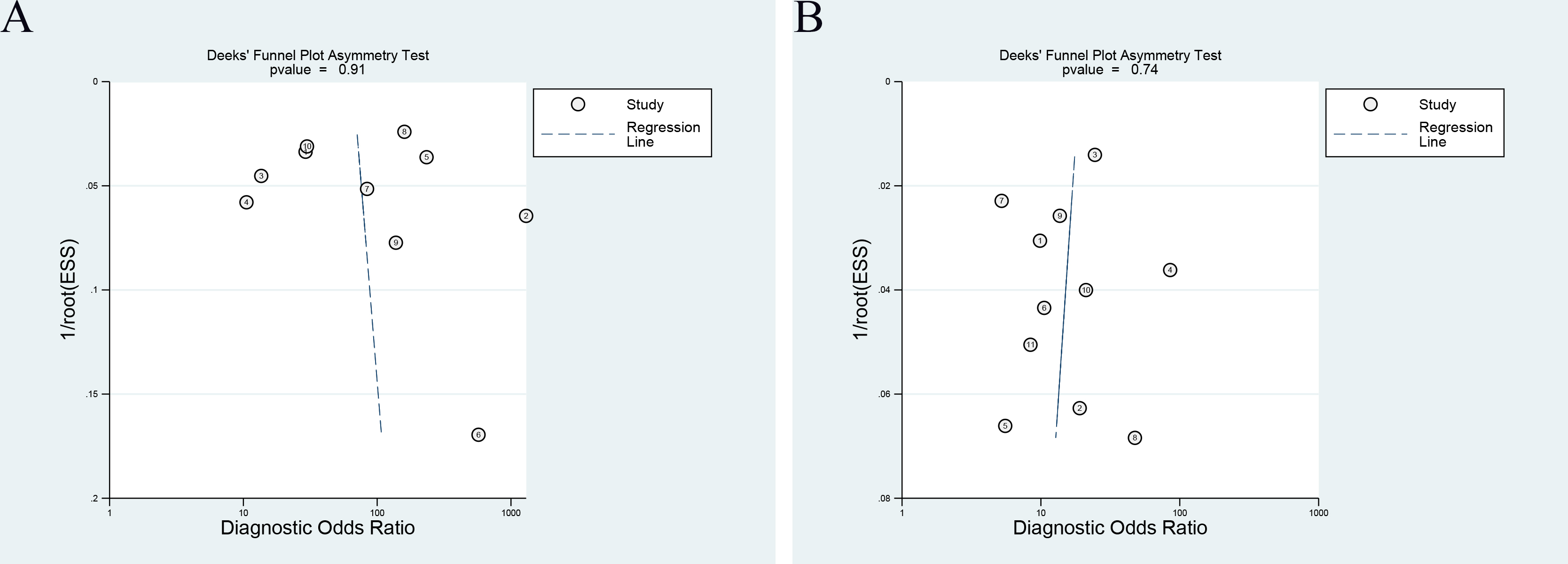

Supplement: Supplementary Figure 2 — Deeks funnel plot. (A) Assessing publication bias in study entities evaluating clinical pregnancy and (B) assessing publication bias in study entities evaluating live birth. [file Image2.jpeg]
